# State of Digital Education Options in the areas of Medical Terminology and the History, Theory and Ethics of Medicine

**DOI:** 10.3205/zma000959

**Published:** 2015-05-13

**Authors:** Maximilian Schochow, Florian Steger

**Affiliations:** 1 Martin Luther University Halle-Wittenberg, Institute for History and Ethics of Medicine, Halle/Saale, Germany

**Keywords:** History, Theory and Ethics of Medicine, Digital Education, Forms of Cooperation, E-Learning, Curriculum, Medical Terminology

## Abstract

**Background:** Institutes of the history of medicine, the theory of medicine, and medical ethics at German institutions of higher learning have created various e-learning options that are based on different learning platforms and tailored to the specific curricular needs of individual teaching. Up to now no valid data has been available about the types of such e-learning options as well as possibilities of future developments thanks to coordinated cooperation among the different institutes.

**Methods:** Of 31 German institutes of the history and theory of medicine and medical ethics that were asked to fill out a questionnaire, 30 answered, which equals a return rate of 97 per cent. The questionnaire was completed between July and August 2012 using a telephone survey.

**Results: **Available to students online, digitally interactive teaching tools have boomed in the course of the last few years at German institutes of the history of medicine, the theory of medicine, and medical ethics. This trend is also reflected in a willingness of more than half of the respective departments (67 per cent) to expand their e-learning options on the basis of previous experience. The offered e-learning systems are accepted very well by the students. 57 per cent of the institutes stated, that 90-100 per cent of the students use the offered systems regularly. E-learning courses for terminology are offered particularly often, this is also reflected in the intended extension of these courses by the majority of institutes which plan to expand their e-learning systems.

**Conclusions: **This article discusses the results of a comprehensive empirical survey about e-learning. It illustrates ways in which individual German institutes plan to expand their e-learning options in the future. Finally, specific proposals for cooperation among institutions (not just online) are introduced, the purpose of which is to produce synergy in e-learning.

## Zusammenfassung

**Hintergrund:** In Deutschland haben viele Einrichtungen für die Bereiche Geschichte, Theorie und Ethik der Medizin E-Learning-Angebote produziert, die auf unterschiedlichen Lernplattformen basieren (LMS) und spezifisch auf die jeweilige curriculare Präsenzlehre zugeschnitten sind. Bisher existieren keine validen Daten darüber, welche Einrichtungen E-Learning-Angebote bereitstellen und welche Formen des E-Learning diese anbieten.

**Methoden:** Von den 31 deutschen Einrichtungen für Geschichte, Theorie und Ethik der Medizin, die gefragt wurden, den Fragebogen auszufüllen, haben 30 geantwortet, was einer Rücklaufquote von 97% entspricht. Die telefonische Befragung fand zwischen Juli und August 2012 statt.

**Ergebnisse: **Der Einsatz digitaler interaktiver Lehrmaterialien, die webbasiert den Studierenden zur Verfügung gestellt werden, hat in den vergangenen Jahren eine Konjunktur an deutschen Einrichtungen für die Bereiche Geschichte, Theorie und Ethik der Medizin erfahren. Dieser Trend wird sich weiter fortsetzen, da mehr als die Hälfte (67%) der Einrichtungen ihre bisherigen E-Learning-Angebote ausbauen will. In den vergangenen Jahren sind vor allem Angebote für Medizinische Terminologie entstanden. Viele Einrichtungen möchten künftig ihre Angebote für Medizinische Terminologie weiterentwickeln. Insgesamt wurden die E-Learning-Angebote von den Studierenden sehr gut aufgenommen. 57% der Einrichtungen gaben an, dass 90-100% der Studierenden die Angebote regelmäßig nutzen.

**Schlussfolgerungen: **Dieser Artikel diskutiert die Ergebnisse einer umfangreichen empirischen Studie über E-Learning. Er zeigt den Rahmen auf, in dem deutsche Einrichtungen vorhaben, ihre E-Learning-Optionen in der Zukunft auszuweiten. Schließlich werden spezifische Vorschläge für die Zusammenarbeit unter den Einrichtungen (nicht nur online) gemacht, deren Ziel es ist, Synergien im Bereich E-Learning zu erzeugen.

## I. Formulating the Question

Over the last few years e-learning has flourished and it is no longer possible to imagine institutions of higher learning at home and abroad without it [1]. As a result, it is almost impossible to keep track of international publications on the subject. Even in national contexts, it is increasingly difficult to maintain an exact overview of developments especially in specific, subject-related contexts [2], [3]. Numerous monographs, collected essays, and journal articles in the area of medicine have introduced questions about the development of innovative e-learning concepts that demonstrate its potential and give suggestions for the institutionalization of e-learning in departments of medicine [4]. Besides these multiple publications, the coming of e-learning or blended learning is also reflected in multi-media e-learning modules that have by now been established in medical faculties and university clinics across Germany. Examples include the e-learning competence centre at the Charité in Berlin or HaMeel, which is the e-learning initiative at the medical faculty of the Martin-Luther-University Halle-Wittenberg.

In the fields of history, theory, and ethics of medicine, however, the picture with regard to e-learning is fragmented [5], [6]. In the respective German institutes, the introduction of digitally interactive online teaching tools, led to increased popularity of such e-learning options with students [7]. In the age of web 2.0, this development is hardly surprising, and the benefits of e-learning are obvious: supporting and expanding curriculum-based, individual teaching; stimulating independent learning; increasing motivation; and being able to check on the level of knowledge.

While in recent years some reports in the field of medical ethics have given insight into the possibilities and limitations of e-learning [8], [9], such reports are missing from the areas of medical history and theory [10]. In 2008, a first such discussion took place during a symposium of the Medical History Association (Fachverband Medizingeschichte e.V.) on the subject of “e-learning in the cross-sectional area of history, theory and ethics of medicine and in medical terminology” [http://www.fachverband-medizingeschichte.de/5bildung/2008_Einladung_Workshop_elearning.pdf]. Since then, it has become obvious that each e-learning option was specifically tailored using different learning platforms (e.g. Moodle, ILIAS).

This realization was the starting point of our study, which asked (1) about the status of e-learning options at German institutes of the history and theory of medicine as well as medical ethics. From this, an up-to-date overview of the numbers, types, and forms of each option was derived. We furthermore (2) examined if – and, more specifically, how – the individual digital learning options should be interconnected in the future, as well as which objectives the individual institutions pursued regarding e-learning. In order to be able to use the possible synergy effects of a coordinated collaboration between the individual institutes, technical prerequisites and experiences had to be considered (3). As a result of this study, we developed a nationwide map of e-learning at German institutes of the history and theory of medicine and medical ethics, which is based on most recent data besides exploring possibilities of cooperation among institutes with regard to e-learning.

## II. Methods

The study is based on an anonymous questionnaire, in which e-learning was construed as an open term. We define the expression “e-learning” as including all forms of learning “in which electronic or digital media are employed for the presentation and distribution of teaching material and/or the support of interpersonal communication” [11], [12]. We only offered the questionnaire to German institutes of the history and theory of medicine and medical ethics that are affiliated with a department of medicine. We chose this criterion because such institutes offer teaching and education.

The questionnaire contained open-ended questions, yes-no questions and “classification” questions. The questionnaire included 29 items and was divided into three sections: The first section included matters of how generally to use e-learning (implementation, time of implementation, types of e-learning, future planning for e-learning Options). The second section contained items on infrastructure and staffing (Learning Management Systems, Content Management Systems, authoring tools, creation of e-learning options). The last section included items on user behavior (students, employees, forms of cooperation).

All items were pretested to reduce confounding factors and optimize its validity. Data were collected from July to August 2012 resorting to a telephone survey. The advantage of this particular survey method lies in the relatively high response rate. Moreover, misunderstandings because if incorrect completion of the questionnaire can more easily be avoided [13].

## III. Results

### Return Rate, Implementation, and Time of Implementation

Of the 31 German institutes of the history and theory of medicine and medical ethics, 30 participated in the telephone survey, which equals a return rate of 97 per cent. 26 of the 30 institutes that participated offer e-learning options to their students, which make up an integral part of the curriculum. The four institutions that did not have e-learning were not planning on implementing e-learning in the future, either. Initial approaches to establishing e-learning options can be traced back to the early 2000s. Between 2002 and 2007, between one and two initiatives were launched per year. The vast majority, that is, seven institutes, began to provide digital learning options in 2008, the year of the above-mentioned Medical History Association’s symposium.

#### Implemented E-learning

In order to establish how different types of e-learning were implemented, we listed e-learning options for a variety of possible courses in the questionnaire. We found for courses on medical terminology, institutions frequently provide materials, a chat room, or vocabulary trainers to accompany the course. At some institutions, dental terminology is offered as an extra course alongside medical terminology. We evaluated these institutions separately (see table 1 (Tab. 1)).

For electives in the first and second stages of the course, institutes for the most part offer a collection of materials as well as chat rooms and forums to accompany the course (see table 1 (Tab. 1)). In cross-section Q2 “History, Theory and Ethics of Medicine” and cross-section Q7 “Medicine of Aging” institutes frequently provide various materials online. A similar picture emerges with regard to cross-section Q13 “Palliative Medicine”, for which five institutes offer e-learning materials and one even e-exams. These comparatively low numbers may be explained by the fact that many institutions do not yet provide any Q7 or Q13 courses. It also remains to be seen in what ways options for Q13 will develop when the new Q14 “Pain Medicine” is established (see table 1 (Tab. 1)).

As regards additional e-learning options, the questionnaires revealed grammar exercises (2), practice exam on Q2 “History, Theory and Ethics of Medicine” (1), material on the ethics of animal testing (1), instructions about the online platform (1), questions about seminar texts (2), chat thread on facebook-group (1), online agreement about lecture topics (1), and, last but not least, development of wikipedia entries (1).

#### Future Plans for E-learning Options

**Figure 1:**
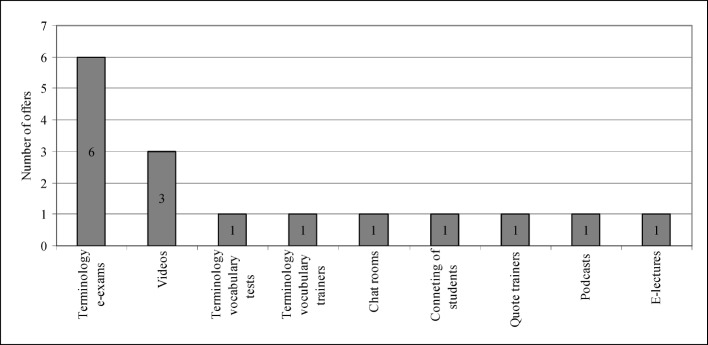
Future planning of e-learning options

While six institutes that were questioned did not plan on expanding what they have, 20 intend to provide more e-learning options in the future. Of these 20, 14 already had specific plans. Some objectives include plans to offer terminology e-exams (6) or plans to offer vocabulary tests and trainers (2). These goals should have been reached by the end of 2013 (see figure 1 (Fig. 1)).

#### Infrastructure

The second part of the questionnaire comprised questions on infrastructure and staffing. Above all, the platforms’ technologies is of interest in this context, because not all platforms are compatible, which presents a crucial barrier for sharing e-learning options. As, for instance, the Learning Management System (LMS) does not match the Content Management System (CMS) at all institutes, we asked about both systems [14].

**Figure 2:**
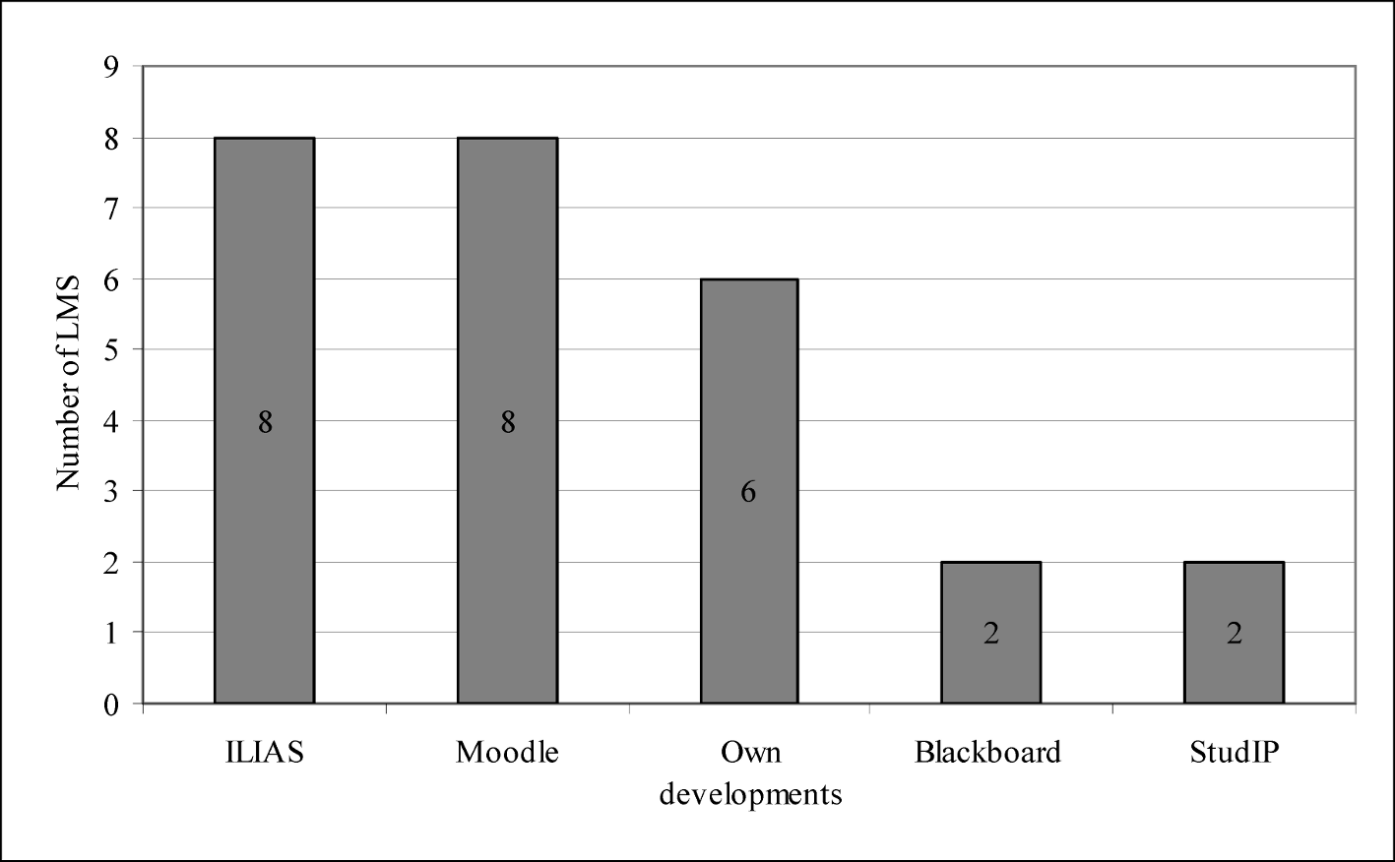
Learning Management Systems (LMS)

**Figure 3:**
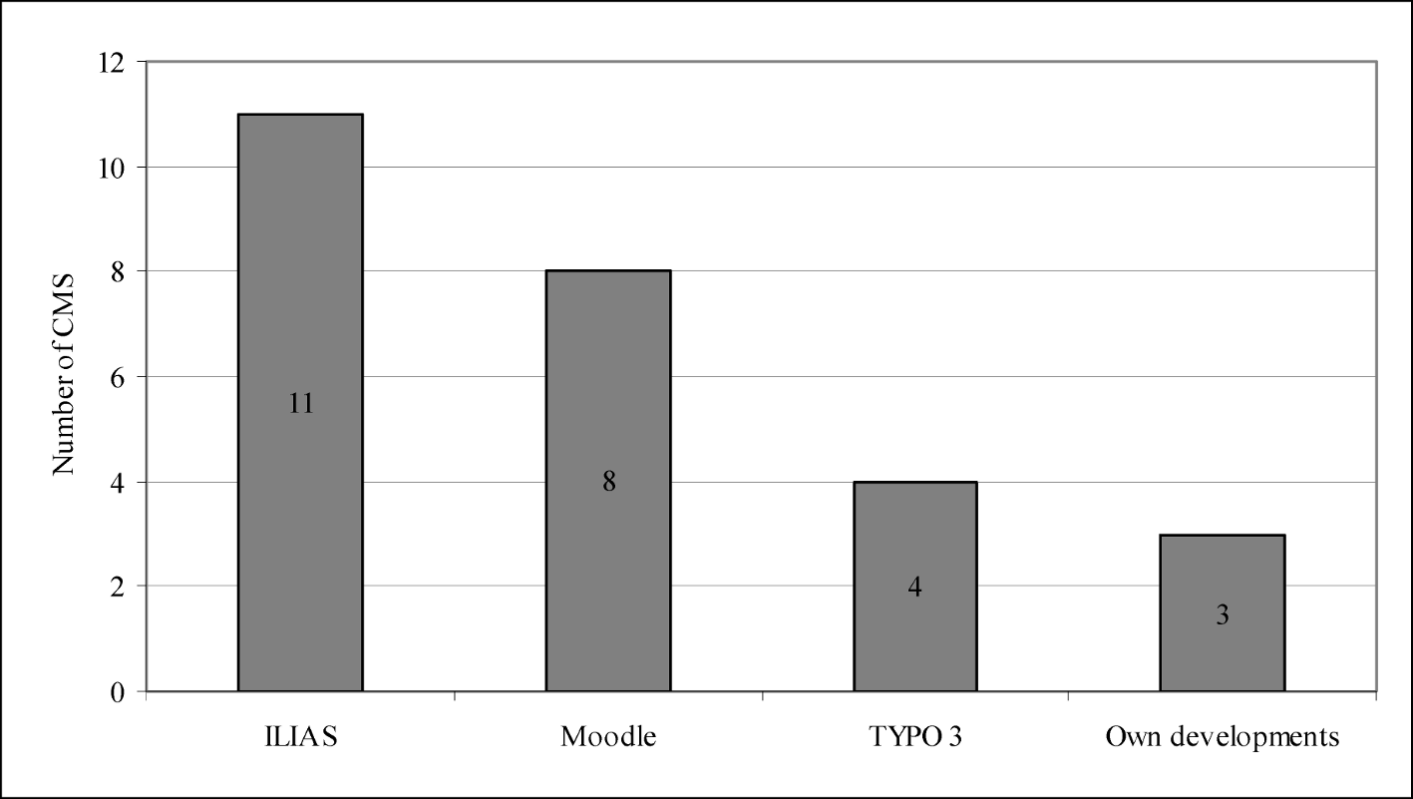
Content Management Systems (CMS)

We discovered that institutions mostly use Moodle or ILIAS as Learning Management System (see figure 2 (Fig. 2)). Regarding the Content Management System (CMS), they tend to create e-learning options with Moodle or work with ILIAS (see figure 3 (Fig. 3)). LMS and CMS are furthermore important when it comes to the technical implementation, and so is the so-called authoring tool (AT) with the help of which special usages, such as animations, can be created. Until recently, very few institutions offered videos or complex animations, which is why few of them were able to respond to related questions: three institutions used authoring tools (AT); one institution installed Adobe Authorware, one institution used IMS, and one institution worked with DBT-Express.

#### Creating E-learning Offers

When asked about staff, the institutes revealed that professors, research staff, and student assistants are involved in creating e-learning options. At some institutes the secretarial staff also contributes to creating material. The fact that 22 institutions chose not to have an external company or supplier to install e-learning options supports this picture. Four institutions used temporary external services, with the majority of these work contracts ending during or after the implementation phase. In addition to that, five institutes used part of their annual budget to hire temporary employees who are responsible for reworking, advising, and maintaining the e-learning options. Of these five, two institutes each have a student assistant position, and another two have each two student assistant positions for e-learning.

#### User Behavior

**Figure 4:**
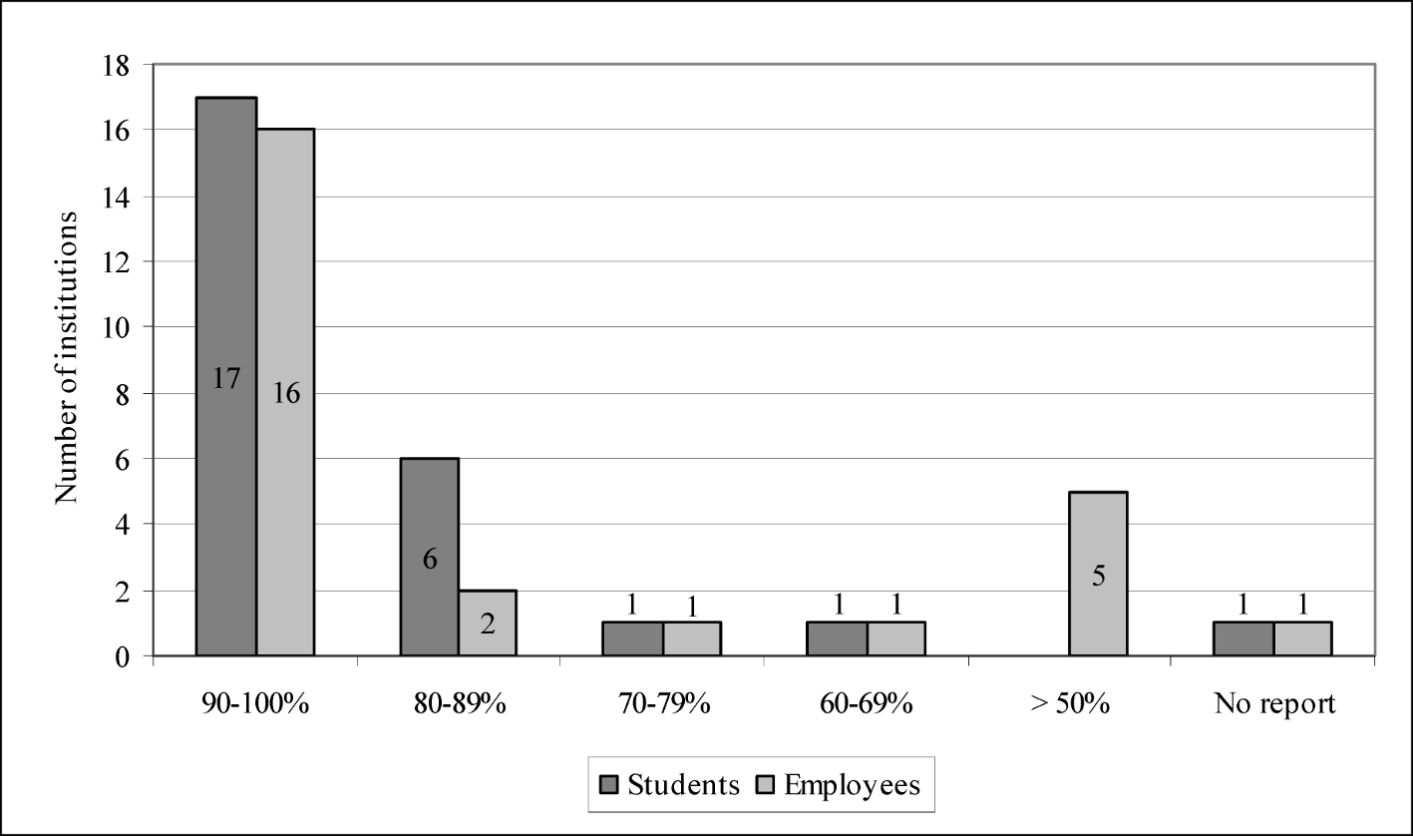
User behavior

User behavior was evaluated in the last section of the questionnaire. 20 institutes said that e-learning options were regularly evaluated, while six had no reliable evaluation system. However, all 26 institutions indicated that e-learning options were revised and updated on a regular basis. Regarding the behavior of student-users, 17 institutions pointed out that 90-100 per cent of their students used the e-learning options. One institute did not report. The information regarding usage on the part of the institutes’ employees was very similar: While one institute neglected to respond, another 16 institutes said that 90-100 per cent of their employees resorted to the e-learning possibilities (see figure 4 (Fig. 4)).

#### Forms of Cooperation

**Figure 5:**
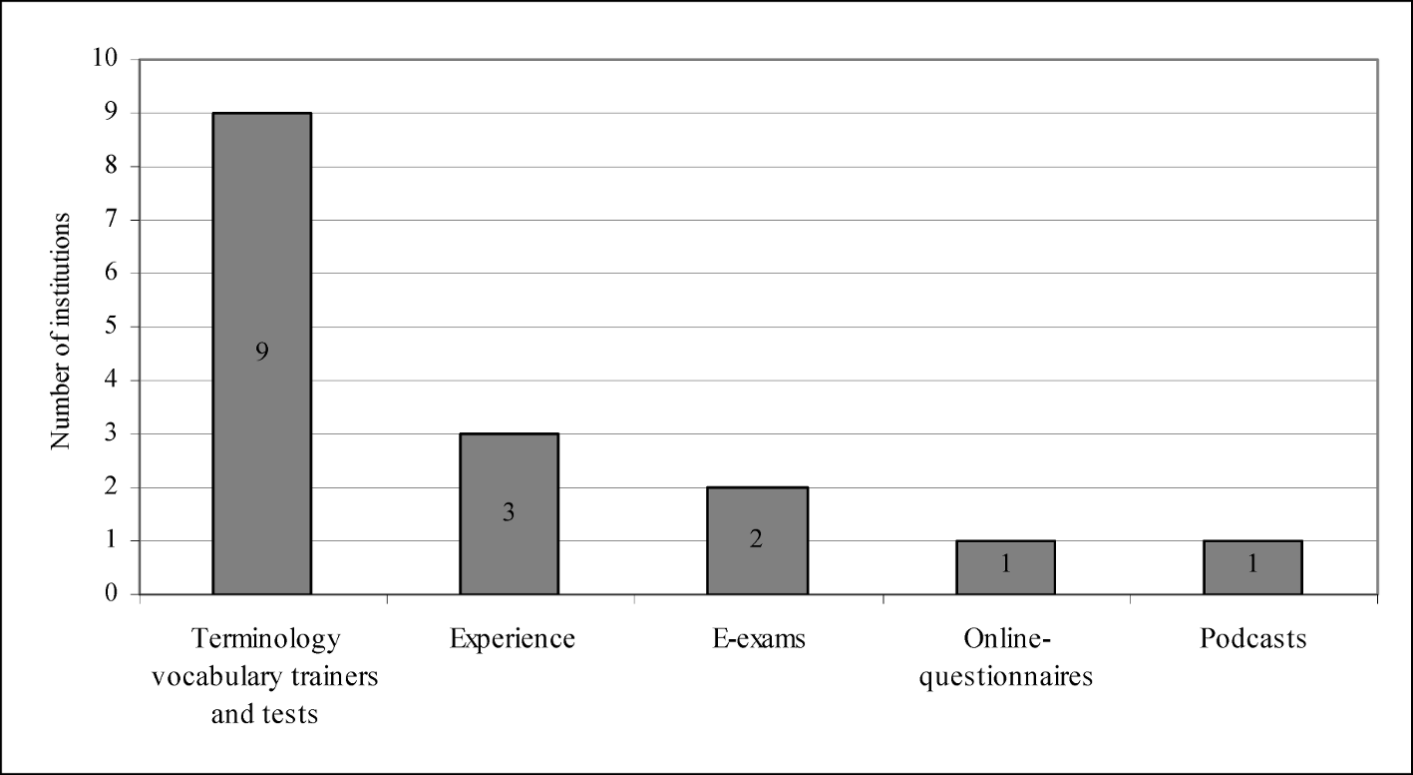
Forms of cooperation between the institutions

Last but not least, we asked if the individual institutes would be prepared to exchange know-how with other German institutes in the areas of the history of medicine, the theory of medicine, and medical ethics. Nearly all institutes agreed to do so, that is, 25 said that they were willing to engage in such an exchange. One institution was unable to provide any information in that regard. The last question dealt with the types of know-how that would be available. Apparently, institutes most frequently want to offer vocabulary trainers and tests (see figure 5 (Fig. 5)).

## IV. Discussion

### Implementation and Types of E-learning

E-learning is not a foreign concept at German institutes of the history and theory of medicine and medical ethics. In fact, e-learning is an integral part of the teaching curriculum at 87 per cent of the institutions that participated in the survey. There are a number of reasons for this high quota. Firstly, it is related to the various recent e-learning initiatives in different subject areas at German medical faculties. For example, the HaMeel (Hallensian Medical e-Learning) program was initiated at Martin-Luther-University Halle-Wittenberg. In some cases, a system of performance-based allocation of funding (Leistungsorientierte Mittelverteilung, LOM) was used as an additional impetus to motivate the medical faculties. Secondly, the Medical History Association’s initiatives played an important role. Lastly, the overall positive outcome of the survey may be explained by our relatively broad definition of the term e-learning.

If the term e-learning had been limited to specific ideas such as multi-media, multi-codification or multimodality, a different overall picture might have emerged. For example, when only interactive teaching and learning options are counted, the number of institutes with e-learning options would rise to 53 per cent, as 16 of 30 institutes offer at least one interactive teaching or learning option (vocabulary trainer and/or tests for medical terminology, grammar trainer and/or tests for terminology, e-exams), in addition to material accompanying the course. A mere ten of the 30 institutes—i.e., 33 per cent of all—only have non-interactive, password-protected material to supplement courses on their learning platforms. Of these ten institutions, five intend to develop an interactive teaching and learning option to offer their students by 2013. They already have specific plans ranging from e-exams to vocabulary trainers or tests. The other five institutes want to keep the status quo and solely offer material to accompany courses.

#### Future Planning of E-learning Options

Our research showed that 67 per cent of the institutes that participated in the survey wanted to expand their e-learning options in the future. The majority of these institutes should have implemented specific plans by 2013. Resorting to chat rooms, videos, and podcasts, the general emphasis was on the implementation and expansion of medical terminology. Vocabulary and grammar trainers were an upcoming trend. This tendency is confirmed by the current situation: Up to now, much time and money has been invested into building and expanding e-learning options in the area of medical terminology, for which we now have vocabulary trainers and tests, chat rooms, podcasts, and e-exams.

A large number of institutions with e-learning options—as well as an increasing number of institutions that want to provide more such options in the future—insinuates that skepticism regarding e-learning is disappearing. This growing acceptance is also evident in the fact that lecturers and students frequently use these options. Even though much of the data that is uploaded onto the learning platforms is used as exam preparation, it is obvious that e-learning presents us with possibilities to provide students with more knowledge and alternative forms of teaching and studying. Concerns are diminishing on the part of the teaching staffs that jobs might be lost and that individual achievements would no longer be transparent in an e-learning scheme. Instead, the staffs have developed more positive attitudes towards experimenting with new possibilities of teaching and learning [15].

#### Infrastructure and Cooperation

The question of possible synergy effects thanks to a coordinated collaboration among institutes has two sides. On the one hand, we had to ask about technical prerequisites and experiences. On the other, we dealt with the extent to which the institutions were actually willing and prepared to exchange know-how. In terms of technical requirements, the majority of institutions works with Moodle or ILIAS. To the extent to which these two systems are incompatible, a cooperation between Moodle and ILIAS users is impossible. However, we would like to propose two strategies to enable future cooperation nonetheless; one on a short-term, the other on a long-term basis.

Possibilities of short-term cooperation can be demonstrated with the example of teaching medical terminology: Of the institutes that work with LMS Moodle, seven have options in the area of medical terminology, only three do not offer terminology. The picture regarding LMS ILIAS is similar: seven institutes have e-learning options for medical terminology, four do not. It may be concluded that the institutions with the same Learning Management System (Moodle or ILIAS) could support one another. Likewise, an exchange could take place between institutes with the same LMS that use only a vocabulary trainer or only a grammar test. That is, the vocabulary test could be exchanged for the grammar trainer.

The same holds true for other e-learning options such as podcasts, e-exams, and specialized videos for Q13 “Palliative Medicine”. In the past, universities chose the technical infrastructure (LMS and CMS) without the individual faculties and institutes having a say in the decision-making. As a consequence, institutes of for example the history, theory, and ethics of medicine could not easily exchange their e-leaning options. As a swift alteration of the infrastructure seems unlikely, a parallel structure, consisting of Moodle on the one hand and ILIAS on the other, is expected to become norm in coming years. In the short term, this would require building up possible structure of cooperation within these two systems, which could then be supplemented with cross-system field reports. Such structures might for example encompass fundamental questions about didactics and legal matters regarding the Internet (e.g., copyright).

Possibilities of long-term cooperation might include developing e-learning options independently of Learning Management Systems and Content Management Systems. It would mean not to create e-learning options with the various tools that, for example, ILIAS provides but to design them in a way that they could be compatible with all LMS and CMS. However, this would require a high level of competence in programming language (.xml or .html), which, in turn, would reduce the number of those who create e-learning options. Instead, institutes would have to rely on professional providers in the area of computer science.

Regarding the actual willingness of the institutes to exchange know-how, the results were unanimous: All of them are willing to do so. It remains to be seen to what extent the vocabulary and grammar trainers and tests, or merely design experiences, will indeed be exchanged. One way could be a joint workshop about possibilities and limitations of cooperation in the field of e-learning. Such a setting might also allow participants to determine such possibilities and limitations of e-learning in the specific field of medical history, theory, and ethics. To provide just one example, how could the cross-sections Q7 “Medicine of Aging” and Q13 “Palliative Medicine” compliment one another through e-learning? With the help of videos on topics such as “breaking bad news” and “doctor patient dialogues”, innovative interconnections of different subjects could certainly be realized.

## V. Conclusion

The high e-learning quota of 87 per cent demonstrates that e-learning is an inherent part of the curriculum at German institutes of the history of medicine, the theory of medicine, and medical ethics. Even when the term e-learning is broadened to include interactivity, over 53 per cent of all institutes provide such options for their students. The trend is also reflected in a willingness (67 per cent of the institutes) to expand options on the basis of previous experience.

Despite technical limitations, possibilities of short-term cooperation are diverse, although on account of technical circumstances, they remain limited in the foreseeable future. A short-term alternative could be to create parallel structures, which should be used, firstly, because the acceptance and usage of e-learning options among students is high, and, secondly, because a tendency to share e-learning resources may be traced, a process that is supported by external funding. The development is illustrated by a collaborative project funded by the German Federal Ministry of Education & Research: “Practical Clinical Competence. Network for Methodological, Didactical and Curricular Optimization of the Study of Human Medicine” [16]. The first of its three sections (i.e., the methodological section) aims to connect universities for mutual application of electronic visual content.

Finally, it is also necessary to check possibilities of cooperation in the long run, such as those that would be independent from specific platforms in order to enable a simpler exchange among institutes in the future. These and other issues should be dealt with at meetings organized by German institutes in the areas of the history of medicine, the theory of medicine and medical ethics. Issues to be discussed might include options that are being or have already been prepared by the institutes in their skills labs and elsewhere. Central questions could include which options can be developed for general use in history, theory, and ethics of medicine, and which standards they would be expected to reach.

## Take-Home-Message

- E-learning is a part of the curriculum that is put to use at German universities in the areas of the history of medicine, the theory of medicine, and medical ethics.
- In the areas of the history of medicine, the theory of medicine, and medical ethics, German universities frequently offer e-exams, chat rooms and forums besides electronic opportunities to practice and test medical vocabulary.
- Despite technical limitations for the time being, the possibilities of short-term cooperation among institutes are numerous.

## Competing interests

The authors declare that they have no competing interests.

## Figures and Tables

**Table 1:**
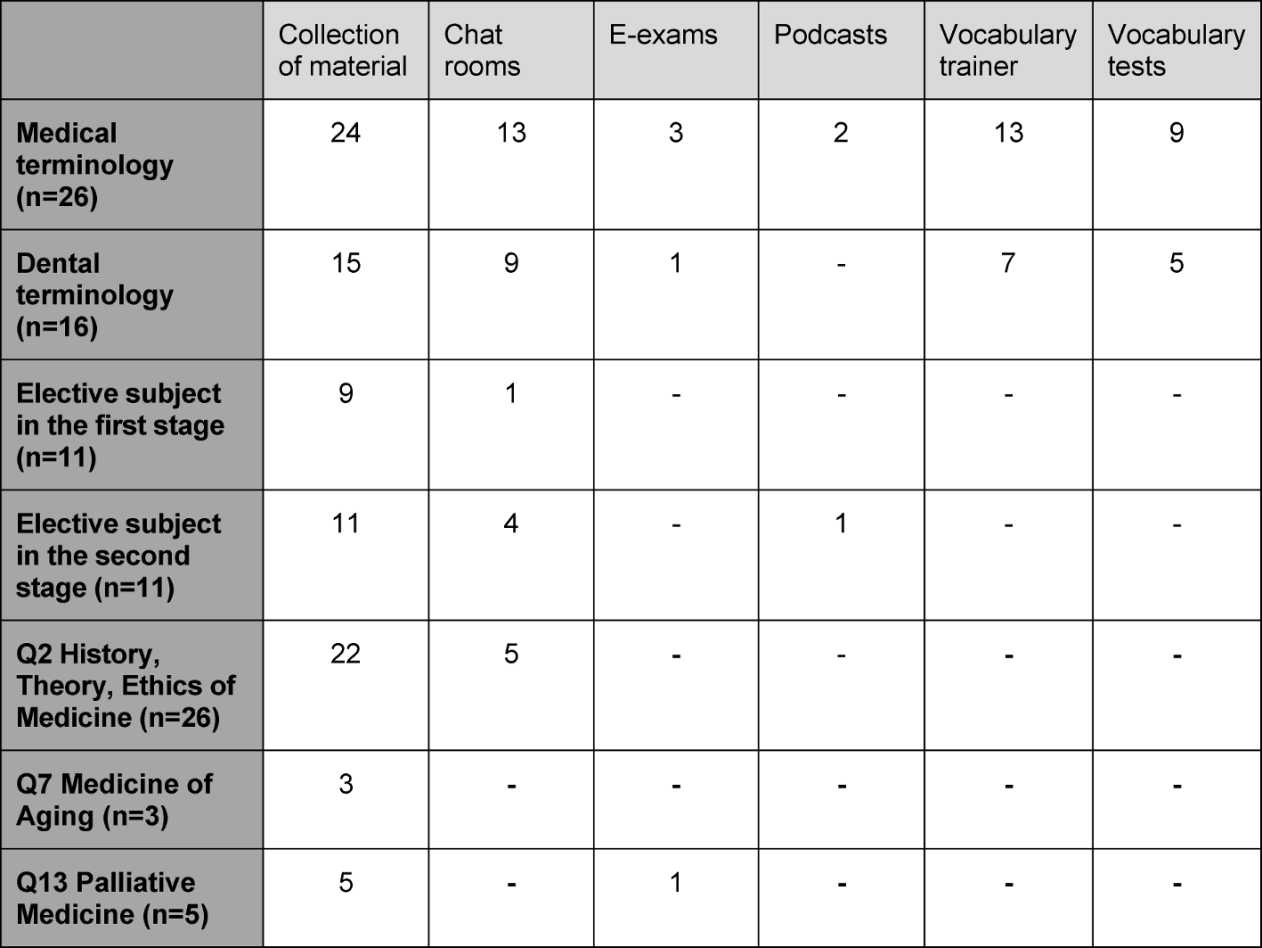
Courses and implemented types of e-learning options (numbers of institutions)
